# 

**Published:** 1905-03-11

**Authors:** 


### NOTICE TO CORRESPONDENTS.

All MSS., Letters, Books for Review, and other matters intended for the Editor should be addressed The Editor, "The Hospital," The Hospital Buildings, 28 & 29 Southampton Street, Strand, W.C.

The Editor cannot undertake to return rejected MS., even when accompanied by stamped directed envelope.

**Notice to Advertisers and Subscribers.**

All Advertisements, Orders for Copies of The Hospital, and Business Communications should be addressed to **THE MANAGER (Not to the Editor),** The Hospital, 28 & 29 Southampton Street, Strand, London, W.C.

The Cost of Subscription, post free, is as follows:—Per week, 3½d.; per quarter, 3s. 3d.; per half-year, 6s. 6d.; per annum, 13s. Foreign and Colonial:—Per annum, 19s. 6d., payable, in all cases, in advance.

**NOTICE.—Vol. XXXVI. of "The Hospital" (April to September 1904), in handsome cloth binding, is now on sale at the office of this Journal, price 10s. 6d. Cases for binding the Numbers contained in this Volume 2s. Index to Vol. XXXVI., 3½d., post free.**

**The Monthly part for March is now ready. Price 1s., by post, 1s. 3d.**

### Medical Appointments.

**William B. Addison**, M.B. B.C., M.R.C.S., L.R.C.P.Lond., Medical Referee under the Workmen's Compensation Acts, 1897 and 1900, and to act for Croydon and Epsom County Courts in County Court Circuit No. 45. **G. F. Aldous**, F.R.C.S.Edin., Assistant Surgeon to the East Cornwall Hospital, Plymouth. **C. B. Blackburn**, M.D.Syd., Honorary Pathologist to the Royal Alexandra Hospital for Sick Children, New South Wales. **J. Bradley**, Certifying Surgeon under the Factory and Workshop Act for the Gortin District of the county of Tyrone. **P. L. Broadbent**, M.B.Syd., Junior Medical Officer in the Lunacy Department, New South Wales. **H. W. Cooper**, M.D., Government Medical Officer and Vaccinator at Wyong, New South Wales. **E. L. Fox**, M.D.Cantab., Physician to the East Cornwall Hospital, Plymouth. **A. W. Graham**, M.B.Lond., M.R.C.S., L.R.C.P.Lond., Port Health Officer at Beauty Point, Tasmania. **O. C. Gruner**, M.B.Lond., Honorary Pathologist to the Leeds Public Dispensary. **C. R. Killick**, M.B.Lond., L.S.A., Certifying Surgeon under the Factory and Workshop Act for the Williton District of the County of Somerset. **R. H. Lucy**, M.B., C.M.Edin., Surgeon to the East Cornwall Hospital, Plymouth. **George Gavain Morrice**, M.A., M.D.Camb., M.R.C.P., Honorary Physician to the Dorset County Hospital. **Thomas William Myles**, B.A., M.B., B.Ch.Irel., R.N., Surgeon to the Plymouth Division of Royal Marines (temporary). **J. M. Pardey**, M.B., Ch.B., Honorary Medical Officer to the Launceston Hospital, Tasmania. **D. Wells Patterson**, M.B., B.S.Durh., Honorary Physician to the Hospital for Diseases of the Skin, Newcastle-upon-Tyne. **G. Stevens Pope**, L.R.C.P.&S.Edin., L.F.P.S.Glas., Medical Superintendent of the Somerset and Bath County Asylum, Wells, Somerset. **J. J. Prendergast**, M.D.Dub., Local Health Officer at Strahan, Tasmania. **C. E. Russell Rendle**, L.R.C.P.Lond., M.R.C.S., Assistant Surgeon to the East Cornwall Hospital, Plymouth. **E. G. Smith**, M.R.C.S., L.R.C.P.Lond., Anæsthetist to Out-patients at the East Cornwall Hospital, Plymouth. **Bertram Soltau**, M.D.Lond., Physician to the East Cornwall Hospital, Plymouth. **H. W. Webber**, F.R.C.S.Edin., Assistant Surgeon to the East Cornwall Hospital, Plymouth. **C. Hamilton Whiteford**, M.R.C.S., L.R.C.P.Lond., Anæsthetist to In-patients at the East Cornwall Hospital, Plymouth. **W. Crosbie Whiteford**, Anæsthetist to In-patients at the East Cornwall Hospital, Plymouth. **C. Whipple**, L.R.C.P.Lond., M.R.C.S., Surgeon to the East Cornwall Hospital, Plymouth. **Robert Wilmott**, F.R.C.S.-Edin., Official Visitor to the Hospital for the Insane, Launceston and New Norfolk, Tasmania. **W. L. Woollcombe**, L.R.C.P.-Lond., M.R.C.S., Surgeon to the East Cornwall Hospital, Plymouth.

### "The Hospital" Institutional Library.

**"Hospitals and Asylums of the World." 4 Vols., with a Portfolio of Plans, complete £12 12s.**

**"Burdett's Hospitals and Charities, 1904." 5s. net.**

**"Burdett's Official Nursing Directory." 8s. net.**

**"Cottage Hospitals, General, Fever, and Convalescent." 10s. 6d.**

**"Uniform System of Accounts for Hospitals and Public Institutions." New Edition. 4s. net.**

**"Hospital Expenditure: The Commissariat." 2s. 6d.**

**"A Legal Handbook for the Use of Hospital Authorities." 2s. 6d.**

**"The Cottage Hospital Case Book or Register of Patients." 10s. and 12s. 6d.**

**All these are published by the Scientific Press, Ltd., and may be obtained through any bookseller, or direct from the publishers, 28 and 29 Southampton Street, Strand, London, W.C.**

### BOOKS THAT NURSES NEED

**ELEMENTARY ANATOMY & SURGERY FOR NURSES.**
By William McAdam Eccles. - - 2/6
Post free

**A HANDBOOK FOR NURSES.**
By Dr J. K. Watson. 5/- net
5/4
Post free

**SURGICAL BANDAGING AND DRESSING.**
By W. Johnson Smith, F.R.C.S. 2/-
Post free

**The NURSES' PRONOUNCING DICTIONARY.**
**Medical Terms and Nursing Treatment.**
By Honnor Morten. Revised by Mary Burdett. 2/-
Post free

**A MANUAL OF PHYSIOLOGY.**
**The Human Body.**
By Professor B. Colton. - 5/-
Post free

**OPHTHALMIC NURSING.**
By Sydney Stephenson, F.R.C.S. - 3/6 net
3/10
Post free

**THE ART OF FEEDING THE INVALID.** New Edition.
With Special Chapter on Food in Tuberculosis and Diseases of the Lungs. 1/6
Post free

**A COMPLETE HANDBOOK OF MIDWIFERY.**
By Dr. J. K. Watson - - - 6/- net
6/4
Post free

**A PRACTICAL HANDBOOK OF MIDWIFERY.**
**For the Practising Midwife.**
By Dr. F. Haultain. - - 6/- net
6/3
Post free

**THE MIDWIVES' POCKET BOOK.**
By Honnor Morten - - - 1/-
Post free

**THE ART OF MASSAGE.**
By Mrs. Creighton Hale. - - 6/-
Post free

**THE MODERN NURSING OF CONSUMPTION.**
By Dr. Jane Walker. - - 1/- net
1|2
Post free

**ELEMENTARY TREATISE ON THE LIGHT TREATMENT.**
By Dr. J. Sequeira. - - - 2/6 net
2/9
Post free

**HOSPITAL SISTERS AND THEIR DUTIES.**
By Eva Lückes - - - 2/6
Post free

### Case-books, Charts, and Note-books.

All requirements of Nurses in this direction are supplied in large and small quantities at the smallest possible price.

**PLEASE WRITE FOR A CATALOGUE OF THE LATEST NURSING TEXT-BOOKS.**

**The Scientific Press, Ltd.,** 28 & 29 SOUTHAMPTON STREET, STRAND, LONDON, W.C.

